# Identification of dihydromyricetin as a natural DNA methylation inhibitor with rejuvenating activity in human skin

**DOI:** 10.3389/fragi.2023.1258184

**Published:** 2024-03-04

**Authors:** Cassandra Falckenhayn, Agata Bienkowska, Jörn Söhle, Katrin Wegner, Guenter Raddatz, Boris Kristof, Dirk Kuck, Ralf Siegner, Ronny Kaufmann, Julia Korn, Sascha Baumann, Daniela Lange, Andreas Schepky, Henry Völzke, Lars Kaderali, Marc Winnefeld, Frank Lyko, Elke Grönniger

**Affiliations:** ^1^ Beiersdorf AG, Research and Development, Hamburg, Germany; ^2^ Institute for Bioinformatics, University Medicine Greifswald, Greifswald, Germany; ^3^ Division of Epigenetics, DKFZ-ZMBH Alliance, German Cancer Research Center, Heidelberg, Germany; ^4^ Institute for Community Medicine, University Medicine Greifswald, Greifswald, Germany

**Keywords:** dihydromyricetin, DNMT1 inhibition, DNA methylation, DNAm age clock, skin, rejuvenation

## Abstract

Changes in DNA methylation patterning have been reported to be a key hallmark of aged human skin. The altered DNA methylation patterns are correlated with deregulated gene expression and impaired tissue functionality, leading to the well-known skin aging phenotype. Searching for small molecules, which correct the aged methylation pattern therefore represents a novel and attractive strategy for the identification of anti-aging compounds. DNMT1 maintains epigenetic information by copying methylation patterns from the parental (methylated) strand to the newly synthesized strand after DNA replication. We hypothesized that a modest inhibition of this process promotes the restoration of the ground-state epigenetic pattern, thereby inducing rejuvenating effects. In this study, we screened a library of 1800 natural substances and 640 FDA-approved drugs and identified the well-known antioxidant and anti-inflammatory molecule dihydromyricetin (DHM) as an inhibitor of the DNA methyltransferase DNMT1. DHM is the active ingredient of several plants with medicinal use and showed robust inhibition of DNMT1 in biochemical assays. We also analyzed the effect of DHM in cultivated keratinocytes by array-based methylation profiling and observed a moderate, but significant global hypomethylation effect upon treatment. To further characterize DHM-induced methylation changes, we used published DNA methylation clocks and newly established age predictors to demonstrate that the DHM-induced methylation change is associated with a reduction in the biological age of the cells. Further studies also revealed re-activation of age-dependently hypermethylated and silenced genes *in vivo* and a reduction in age-dependent epidermal thinning in a 3-dimensional skin model. Our findings thus establish DHM as an epigenetic inhibitor with rejuvenating effects for aged human skin.

## Introduction

Aging is a time-dependent general loss of functionality and fitness across lifespan, and the parallel accumulation of molecular damages, including altered epigenomic regulation is considered one of the key hallmarks of aging (Booth and Brunet, 2016). DNA methylation is a key epigenetic modification that has been associated with many aspects of human health and disease (Bergman and Cedar, 2013). DNA methylation is catalyzed by a conserved class of enzymes that are known as DNA methyltransferases (DNMTs) and that cooperate to establish and maintain DNA methylation patterns during embryonic development and tissue homeostasis (Lyko, 2018). Broadly, DNA methylation patterns are established during early embryogenesis by DNMT3A and DNMT3B, and then maintained by DNMT1. DNMT1 is an essential enzyme that functions to copy methylation patterns from the parental (methylated) strand to the newly synthesized strand after DNA replication (Jurkowska and Jeltsch, 2016).

The combined activity of DNMTs results in widespread methylation of the human genome. However, there are also regions that are characterized by low or absent levels of methylation. For example, CpG islands, which are CpG-dense regions that are often associated with the promoters of housekeeping genes, are usually devoid of methylation (Jones, 2012). Furthermore, gene regulatory elements, such as transcription factor binding sites, often show dynamic and context-dependent methylation levels. Indeed, DNA methylation has been shown to modulate transcription factor binding (Yin et al., 2017), which provides an explanation for how DNA methylation modulates gene expression (Schübeler, 2015).

While the role of DNA methylation in mammalian cell fate-specification is now widely accepted (Smith and Meissner, 2013), its role in environmental adaption has been more difficult to substantiate. In this context, the analysis of human skin can be considered particularly relevant, as skin represents the most environmentally exposed human organ and its phenotype is intricately linked to epigenetic regulatory mechanisms (Köhler and Rodríguez-Paredes, 2020). For example, we have shown that skin aging is associated with defined DNA methylation changes, especially hypermethylation (Gronniger et al., 2010; Bormann et al., 2016). A detailed comparison of single-base resolution epidermis methylomes also revealed age-related DNA methylation changes in gene regulatory regions (Raddatz et al., 2013). This provided a mechanistic explanation for how altered DNA methylation in old skin could affect skin phenotypes. Finally, we also showed that age-related methylation changes in the human epidermis can be used to predict the chronological age of sample donors and used these findings to establish an epidermis DNA methylation clock (Bormann et al., 2016). Together, these studies comprehensively illustrate the role of altered DNA methylation patterns in human skin aging.

It has been suggested that healthy lifestyle, which is based on the avoidance of environmental stressors such as UV light and smoking, but also intake of dietary antioxidants, may improve skin appearance through epigenetic modulation (Alegría-Torres et al., 2011). Improved epigenetic plasticity through a moderate inhibition of the methylation pattern could plausibly support this process. As DNA methylation needs to be maintained through an active mechanism, it can be modified by inhibiting the corresponding enzymes, most notably DNMT1. We hypothesize that a moderate reduction of DNMT1 activity might result in a reduction of age-dependent hypermethylation and promote the restoration of the epigenetic pattern to the ground-state. This provides an attractive strategy for identifying new anti-aging compounds.

We have now screened a library of natural substances to identify active compounds that inhibit DNMT1 in a biochemical assay. This identified dihydromyricetin (DHM), a compound from plants that are popular in traditional medicines that is already known for its beneficial anti-cancer, antioxidant, and anti-inflammatory properties (Kang et al., 2011; Zhang et al., 2011). Further analyses showed that this nutritional flavonoid inhibited DNA methylation in cultured primary human keratinocytes and induced a reduction in DNA methylation age, which is an epigenetic predictor of the skin aging phenotype. Additionally, the beneficial modulation of the methylation pattern by DHM is reflected by a reactivation of the expression of marker genes for age-related epigenetic gene silencing *in vivo*. Finally, our results show that DHM has rejuvenating effects in human skin models, which indicates considerable potential for cosmetical applications.

## Results

### Identification of DHM as a natural small-molecule DNMT inhibitor

**FIGURE 1:**
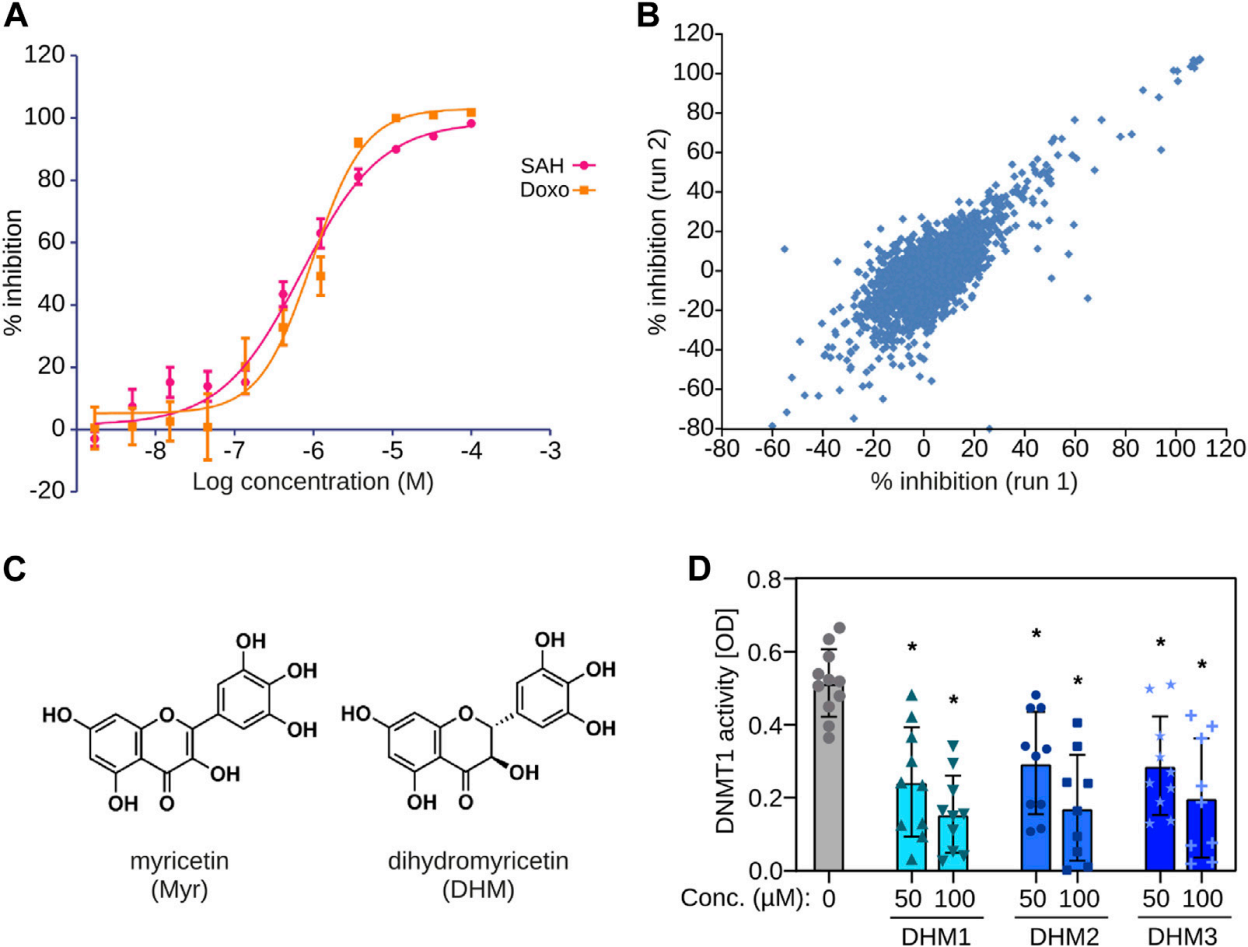
Identification of dihydromyricetin (DHM) as a DNMT inhibitor. **(A)** Establishment of a biochemical assay with human recombinant DNMT1. The plot shows dose-response curves for S-adenosyl-homocysteine (SAH) and doxorubicin (Doxo). **(B)** Scatter plot showing results from two independent screens. **(C)** Chemical structures of myricetin (Myr) and dihydromyricetin (DHM). **(D)** Testing of three independent DHM extracts in an independent biochemical DNMT assay (*n* = 10). *P_adj_ < 0.01 (one-way ANOVA with Bonferroni correction).

We used full-length recombinant human DNMT1 to establish a homogenous 384-well scintillation proximity assay (SPA) with a biotinylated 42mer hemi-methylated oligonucleotide that was captured with yttrium silicate (YSi) streptavidin coated SPA beads. This assay showed clear dose-response curves for S-adenosyl-homocysteine, a known DNMT1 product inhibitor, and doxorubicin, a DNA intercalating compound (Figure 1A). We therefore used this assay to screen a library of 1800 natural products and 640 FDA-approved drugs. Two replicate screens were carried out and showed high reproducibility (r = 0.78, Figure 1B). Screening hits contained a number of substances that are known DNA intercalators, such as doxorubicin, as well as known natural compound DNMT inhibitors, such as epigallocatechinol-3-gallate (Fang et al., 2003), and dietary polyphenols like quercetin, which is known for its anti-cancer effect (Kedhari Sundaram et al., 2019). Interestingly, the screen also identified myricetin (Figure 1C) with a mean IC_50_ of 43.37 µM (Supplementary Figure S1A), a common flavonoid found in grapes, berries, fruits, vegetables, and herbs, among the most potent hits. We then obtained three highly (>95%) pure extracts containing the colorless myricetin derivative DHM (3,3′,4′,5,5’,7-hexahydroxy-2,3-dihydroflavanonol, Figure 1C). DHM can be extracted from plants that are being used in traditional medicines (Zhang et al., 2018), e.g., the Vine tea *Ampelopsis grossedentata* (DHM2 and DHM3) and the bark, leaves and flowers of the Wax Myrtle tree (Myrica cerifera, DHM1). Testing of the three extracts in an independent biochemical DNMT assay (see Materials and Methods for details) indicated significant (P_adj_ < 0.01, one-way ANOVA with Bonferroni correction) DNMT1 inhibitory activity for DHM in the similar concentration range like myricetin (Figure 1D; Supplementary Figure S1B) and no significant differences in efficacy between the three tested extracts (one-way ANOVA). Due to its availability, favorable material properties and known tolerability in humans (Zhang et al., 2018), we prioritized DHM for further analyses. Taken together, these finding provide first evidence for DHM being a DNMT1 inhibitor.

### DHM causes DNA hypomethylation in primary human keratinocytes

**FIGURE 2:**
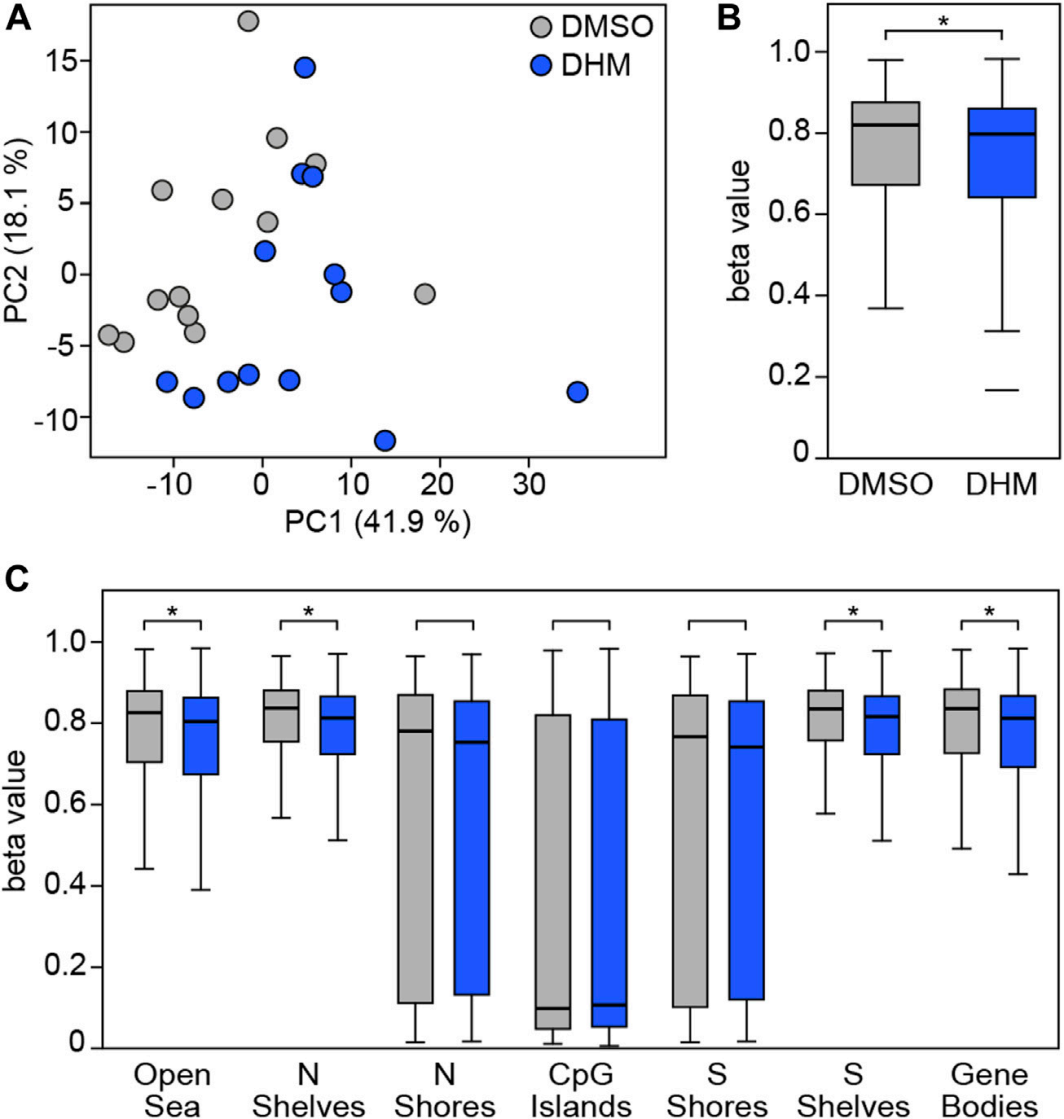
DHM inhibits DNA methylation in primary human keratinocytes. **(A)** Principal component analysis based on 101,067 differentially methylated probes in the complete dataset obtained with primary human keratinocytes from different donors (*n* = 13). **(B)** DHM-dependent hypomethylation in primary human keratinocytes. Box plots indicate the distribution of beta values of the 101,067 differentially methylated probes in the complete dataset. *p = 1.4 × 10^−33^, *t*-test. **(C)** DHM-dependent hypomethylation in epigenomic substructures. *P_adj_ < 6.7 × 10^−4^, *t*-test.

To test the ability of DHM to modulate DNA methylation in human cells, we cultivated 13 independent primary human keratinocyte lines in the presence or absence of the highest tolerable concentration of DHM (Supplementary Figure S2). Subsequently, we isolated genomic DNA from the cultured cells and analyzed genomic DNA methylation patterns using Infinium 850 k arrays. Further data analysis that considered the heterogeneity between individual cell lines identified 101,067 differentially methylated probes (see Materials and Methods for details) that were used for a more detailed analysis. Principal Component Analysis based on the differentially methylated probes showed a detectable separation of DHM-treated and control (DMSO) cells (Figure 2A) which is in agreement with the overall accuracy obtained with random-forest based classification (Supplementary Figure S3), indicating directional effects across all individual cell lines. Further analysis of the differentially methylated probes in all primary human keratinocytes from 13 different donors also showed a moderate, but robust and highly significant (*p* = 1.4 × 10^−33^, *t*-test) reduction of DNA methylation in the DHM-treated cells (Figure 2B). Interestingly, this effect was evident and statistically significant in all epigenomic features, except CpG islands and the surrounding CpG island shores, which represent the features with the lowest baseline methylation levels in the human genome (Figure 2C). Together, these results demonstrate that DHM causes moderate changes of DNA methylation in primary keratinocytes and provide important proof-of mechanism for DHM inhuman cells.

### DHM treatment does not affect general markers of epigenetic and genetic integrity in primary human keratinocytes

**FIGURE 3:**
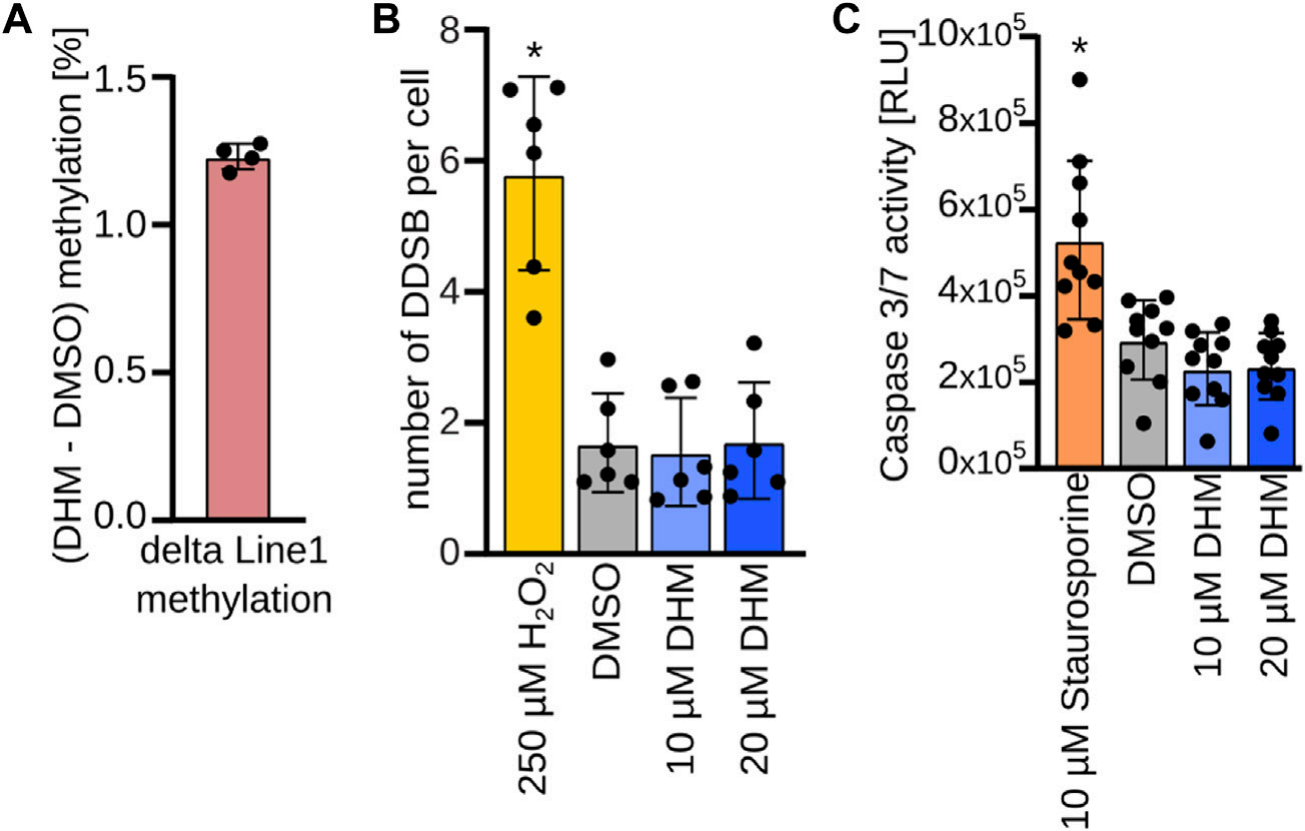
No evidence for critical epigenetic changes upon DHM treatment in keratinocytes. **(A)**. Direct pyrosequencing after bisulfite conversion of the LINE-1 sequence covering methylation of 4 representative CpGs. Bar plot represents mean methylation difference of the analyzed LINE-1 sequence between 20 µM DHM and DMSO treatment in % (*n* = 4). **(B)** DNA damage assay. Bar plots depicting number of DNA double strand breaks per cell in primary human keratinocytes upon DHM treatment (blue) and positive control H_2_O_2_ (yellow) compared to the DMSO control (gray). *p < 0.05 (*n* = 6; 2-way ANOVA). **(C)** Apoptosis assay. Bar plots depicting caspase 3/7 activity in primary human keratinocytes upon DHM treatment (blue) and positive control staurosporine (orange) compared to the DMSO control (gray). *p < 0.01 (*n* = 10; one-way ANOVA, Dunnett’s multiple comparison test).

Profound losses of DNA methylation have been shown to affect epigenetic and genetic integrity, resulting in apoptosis (Yokochi and Robertson, 2004; Laranjeira et al., 2023). We therefore analyzed whether DHM induced global loss of methylation in keratinocytes, using LINE-1 retroelement methylation as an established marker (Zhang et al., 2020). The results showed no reduction of LINE-1 methylation in DHM-treated primary keratinocytes (Figure 3A and Supplementary Figure S4). In addition, we analyzed the development of double-strand breaks after DHM treatment by immunofluorescence staining of gamma-H2AX, an established marker of DNA damage. Our results showed no DHM-dependent increase in gamma-H2AX staining (Figure 3B) in cultured primary keratinocytes from six independent donors. Finally, we investigated whether DHM activates Caspase 3/7 in keratinocytes, which is a widely used marker for apoptosis induction. The results showed no increase in Caspase 3/7 activity after treatment (Figure 3C). Together, these findings suggest that DHM does not affect general markers of epigenetic and genetic integrity in keratinocytes.

### DHM affects epigenetic predictors of the skin phenotype

**FIGURE 4:**
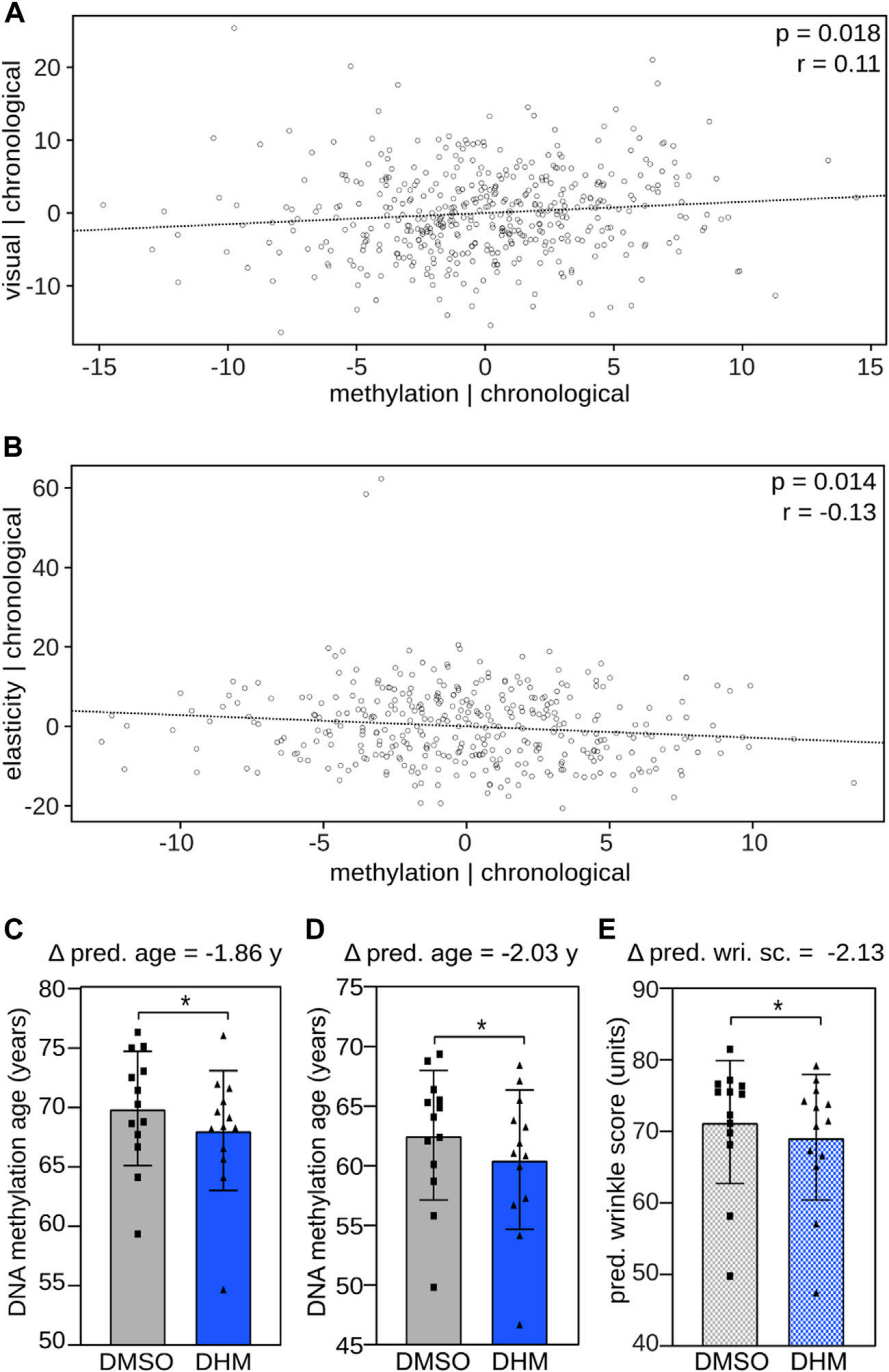
DHM reduces DNA methylation age and predicted wrinkle grade, epigenetic predictors of the skin aging phenotype. Association between DNA methylation age and **(A)** visual age and **(B)** skin elasticity, adjusting for the influence of the chronological age using a skin specific clock (Bormann et al., 2016). Pearson correlation coefficient (r) and significance (p) are depicted. DHM reduces the DNA methylation age of cultured primary human keratinocytes determined **(C)** by the Skin & Blood clock (Horvath et al., 2018) and **(D)** the newly established human epidermis clock. **(E)** Induced reduction of the predicted wrinkle grade upon DHM treatment determined with the recently established wrinkle predictor (see accompanying manuscript by Bienkowska et al.). Every symbol represents an individual cell line from an independent donor. *p < 0.05 (Wilcoxon rank-sum test).

In subsequent analyses, we investigated the relationship between DNA methylation and skin aging phenotypes. In initial analyses, we used a previously established epidermis DNA methylation clock (Bormann et al., 2016) to calculate the epigenetic age of 461 female volunteers from a cohort study (see Material and Methods for details). For the same group of volunteers, we then quantitatively determined their skin aging phenotypes by assessing the visual skin appearance (visual age) and by measuring skin elasticity, respectively. Partial correlation analysis identified a significant (*p* < 0.05, Pearson correlation) positive relationship between epigenetic age and visual age after controlling the influence of the chronological age on both variables (Figure 4A). Similarly, we found a significant (*p* < 0.05, Pearson correlation) inverse association between methylation age and skin elasticity adjusting for chronological age (Figure 4B). These results identify a correlation between epigenetic age and skin aging phenotypes independent of chronological age and establish a suitable framework for evaluating the effect of DHM on epigenetic predictors of the skin phenotype.

Subsequent analyses then addressed the effects of DHM on DNA methylation clocks. To this end, we used 1) the established Skin & Blood Clock (Horvath et al., 2018), 2) a human epidermis clock, which we generated based on data from the 461 female volunteers (Supplementary Figure S5) and 3) a wrinkle predictor, which we recently established by training a model that predicts the wrinkle grade based on DNA methylation profiles of epidermal samples (see accompanying manuscript by Bienkowska et al.). These models were used to analyze methylation patterns from DHM-treated primary human keratinocytes, which reflect the main cell type of the epidermis. Correlating the donor age to the predicted biological age of the cell populations revealed a clear trend, which justifies applying the *ex vivo* developed age clocks to cell culture samples, particularly to identify relative changes after treatment (Supplementary Figure S6). The results showed a consistent and statistically significant (*p* < 0.05, Wilcoxon rank-sum test) reduction in the DNA biological age of around 2 years with both the Skin & Blood clock and with the human epidermis clock (Figures 4C, D). A similar effect was also observed for the predicted wrinkle grade (Figure 4E). Based on the observed correlation between chronological age and observed wrinkle grade (Supplementary Figure S7) this finding indicates a cell rejuvenation by 3.7 years of wrinkle accumulation in female volunteers. Taken together, the applied epigenetic predictors indicate a consistent DHM-dependent cell rejuvenation in the range of 2–3.7 years in keratinocytes.

### *In vivo* and functional effects of DHM on skin aging phenotypes

Before we investigated the effect of DHM *in vivo*, an evaluation of the toxicological profile of DHM was performed to ensure the safe use of the intended cosmetic product application. From our studies, we observed no discernible cytotoxicity at in-use concentrations in toxicological *in vitro* assays with DHM, such as the phototoxicity test (Supplementary Figure S8), which we performed equivalent to the OECD Guideline 498. The safety assessment (see Material and Methods for details) concluded that DHM has a low potential for acute and repeated dose toxicity. Additionally, limits on the concentration of DHM in the formulation have been applied to ensure consumer safety.

To assess the bioavailability of DHM in human skin, we verified that the molecular properties fulfill the criteria that serve as an indicator for a potential skin barrier penetration (Lee et al., 1994; Bos and Meinardi, 2000; Cross et al., 2003; Hadgraft, 2004; Chen et al., 2018): With a molecular weight of 320.25 g/mol and a logP of 1.23 (ChemSpider) dermal absorption into the living skin cells is expected.

**FIGURE 5:**
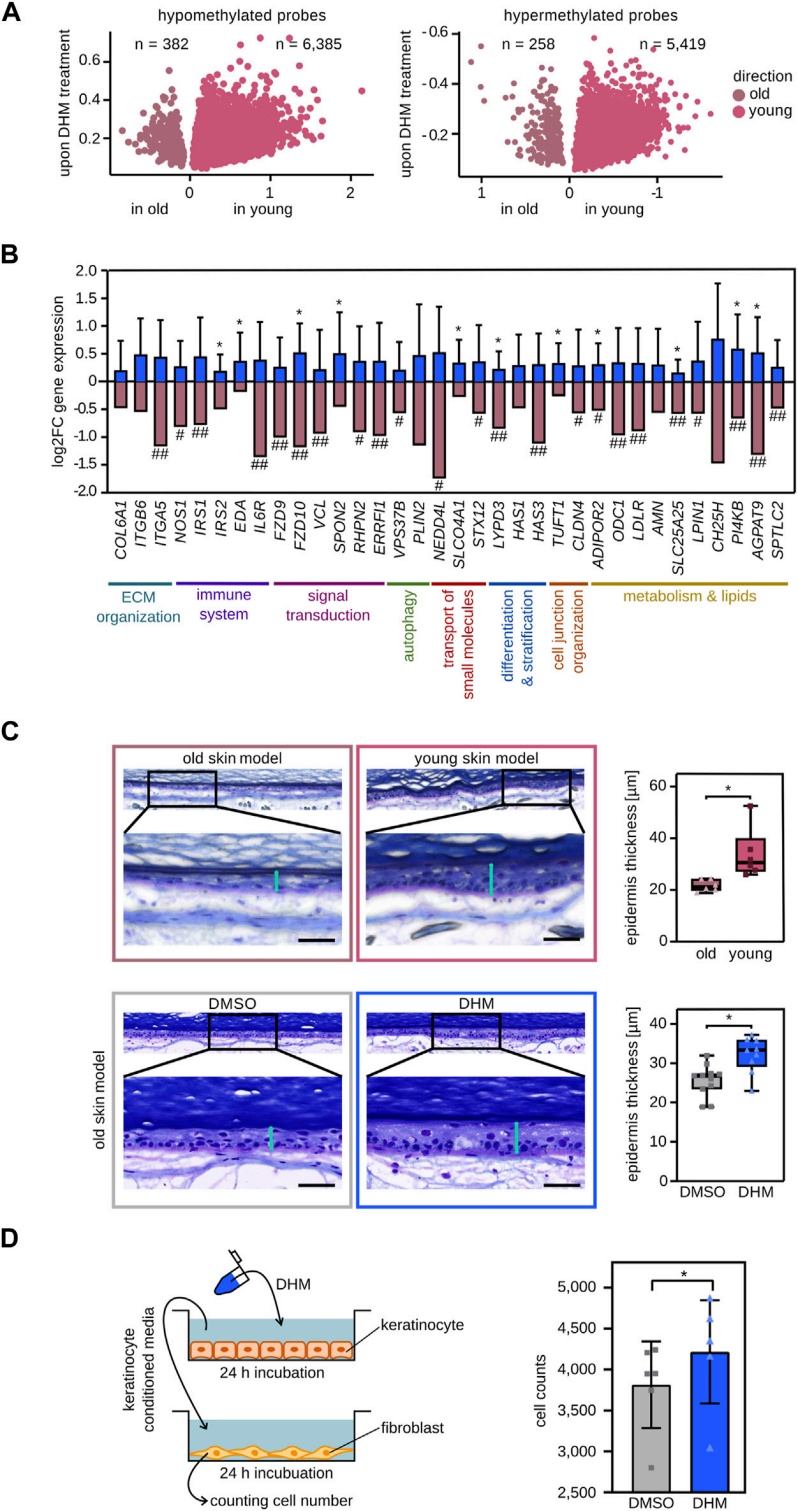
*In vivo* and functional effects of DHM on skin aging phenotypes. **(A)** DHM induces the DNA methylation pattern of the young epidermis *in vivo*. Volcano plot of hypomethylated CpGs upon topically DHM treatment overlapping with hypomethylated CpGs in young (pink) and old (shaded pink) epidermis, respectively (left). Overlap of hypermethylated CpGs after DHM treatment with hypermethylated CpGs in young and old epidermis, respectively (right). **(B)** Topical DHM treatment induces the reactivation of selected marker genes (blue bars) that are subject to age-related epigenetic silencing *in vivo* (shaded pink bars). *p < 0.05 (Wilcoxon rank-sum test). # Significant negative correlation (P_adj_ < 0.05, Pearson correlation with Holm correction) of gene expression with *in vivo* wrinkle grade and ## being among the top 10% of genes negatively correlated with *in vivo* wrinkle grade (see Material and Methods, and Supplementary Figure S9 for details). **(C)** DHM increases epidermis thickness in an organoid skin aging model. Microscopic images of 3D skin models cultivated with primary fibroblasts either form old donors (top left) or from young donors (top middle) and the resulting epidermal thickness measurements (top right). The effect of DHM was analyzed in the same experimental setup and is shown in the lower panels. Vertical turquoise bars: epidermal thickness measurements. Horizontal black bars: 50 μm *p < 0.005 (Wilcoxon rank-sum test). **(D)** DHM increases the proliferation of fibroblasts incubated with DHM-conditioned medium obtained from keratinocytes. Schematic representation of the crosstalk experiment is depicted on the left panel. The effect of DHM on number of counted fibroblasts is shown in the right panel (*n* = 6). *p < 0.05 (Wilcoxon’s signed rank test).

To characterize the effect of DHM *in vivo*, 30 female volunteers were recruited for a vehicle-controlled study where DHM was topically applied to the skin on their lower backs for 2 weeks before receiving a repetitive low-dose UV exposure as an aging stimulus. Subsequently, epidermis samples were obtained from the treated area and their methylation profiles were analyzed. When these profiles were intersected with epidermis methylation profiles from young and old donors (*n* = 204, see Materials and Methods for details), this identified a set of 12,444 age-related DNA methylation probes that were specifically differentially methylated by the DHM treatment. Notably, when compared to vehicle controls, DHM changed the methylation values towards the pattern that is representative of the young epidermis for more than 95% of these probes (Figure 5A). These findings provide an important confirmation for our observations in cultured keratinocytes and suggest an *in vivo* rejuvenating effect on the epigenetic profile of the skin.

To further analyze the effect of DHM on *in vivo* gene expression, 19 female volunteers (50–65 years) were recruited to topically treat their inner forearm with a DHM containing formulation (or with the corresponding vehicle) for 8 weeks. Subsequently, epidermis samples were obtained by suction blistering, RNA was isolated and the expression level of 34 marker genes for age-related epigenetic silencing, of which 23 were associated with wrinkle development *in vivo*, were determined by qPCR (see Materials and Methods and Supplementary Figure S9 for details). The results showed an increased mRNA expression level for all 34 genes in DHM-treated epidermis when compared to vehicle controls (Figure 5B), indicating a substantial capacity for DHM to ameliorate age-related epigenetic gene silencing in the human epidermis.

Finally, to functionally investigate the effect of DHM on skin aging, we established a 3-dimensional skin aging model. To this end, we compared skin models that were grown with primary keratinocytes from a single donor (42 years) and primary fibroblasts of donors from two different age groups (18–33 years and 58–67 years). After cultivating the skin equivalents for 12 weeks, comparison of epidermal thickness showed a significantly (*p* < 0.01, Wilcoxon rank-sum test) thinner epidermis in skin models with fibroblasts from old donors (Figure 5C), which corresponds to a well-known skin aging phenotype *in vivo* (Giangreco et al., 2010). Treatment of old skin models (fibroblasts: 63–68 years, keratinocytes: 23–59 years) with DHM resulted in a significantly (*p* < 0.05, Wilcoxon rank-sum test) increased epidermal thickness (Figure 5C). To further investigate the functional effect of DHM on dermal fibroblasts, we used an experimental setup to analyze epithelial/mesenchymal interactions (Harrison et al., 2005). The results showed that DHM-conditioned supernatant from keratinocytes significantly (*p* < 0.05, Wilcoxon rank-sum test) stimulated fibroblast proliferation (Figure 5D). As decreased proliferation and a reduced cell number of fibroblasts is a marker for skin aging (Varani et al., 2000) these results further support a rejuvenating effect of DHM in cell culture.

Taken together, these *in vivo* and *in vitro* studies provide evidence for a skin rejuvenating effect of DHM on a molecular and functional level.

## Discussion

As natural compounds are well known for their beneficial effects on human physiology, this class of substances represents an attractive resource for the screening of new anti-aging active ingredients. And because altered epigenomic regulation is considered a key hallmark of aging (Booth and Brunet, 2016), the identification of compounds that modulate epigenetic mechanisms is considered a particularly impactful approach to slow down or reverse the process. We have focused our small-molecule screen on natural products and FDA-approved drugs, two classes of compounds with established safety profiles, that do not require further animal testing and tested them for their inhibitory effect on the key epigenetic modulator DNMT1. This approach identified DHM, a flavonoid with known beneficial effects like anti-cancer, anti-oxidative and anti-inflammatory properties. DHM has also been discussed in the context of anti-aging therapy, as it has been shown that DHM protects against cognitive impairment and ameliorates brain aging in rats, as it modulates apoptosis and dysfunctional autophagy of hippocampal neurons, and astrogliosis (Kou et al., 2016; Qian et al., 2021). Additionally, DHM has been shown to activate FOXO and AOP to modulate longevity in *Drosophila* (Fan et al., 2020). Nevertheless, a deep understanding of the mechanisms of action is still missing. Our study could serve as a starting point to investigate how DHM could counteract or prevent aging on a cellular level, even though, our study focused on a specific part of the body, the skin. Beyond the scope of our study, DHM has also been proposed by others to serve as a promising compound to treat human aging conditions (Martínez-Coria et al., 2019). However, there are only few studies that demonstrate the health-promoting effects of DHM in a placebo-controlled manner in humans. As an example, Chen et al. observed after 12 weeks of consuming DHM a decrease in serum TNF-α (Chen et al., 2015), a blood factor which increases with aging (Paolisso et al., 1998; Bruunsgaard et al., 1999). Investigating the potential of DHM to treat aging or age-related diseases systemically would be an exciting approach for future evaluation.

DHM is considered the active ingredient of Vine tea, a health-promoting herbal tea with increasing global popularity and consumption. As DHM and its derivatives are considered to make up 1/5th of dry Vine tea leaves depending on extraction method (Ye et al., 2015; Carneiro et al., 2021), the amount of DHM in a prepared cup of Vine tea can reach mmol concentrations, thus illustrating its excellent tolerability. For topical application of DHM a safety assessment following next-generation risk assessment approaches for new cosmetic ingredients concluded that the substance has only a low potential for acute and repeated dose toxicity, which supports a safe usage up to 0.15% on human skin (see Materials and Methods for details and Supplementary Figure S8). This distinguishes DHM from the most widely used DNA demethylating agents 5-azacytidine and 2′-deoxy-5-azacytidine, which have been developed as clinical anti-cancer drugs targeting aberrant DNA methylation (Jones et al., 2016). However, the broad and indirect mode of action of these hypomethylating agents is associated with noticeable toxicity (Stresemann and Lyko, 2008). More selective small-molecule inhibitors, such as RG-108 (Brueckner et al., 2005) and GSK3685032 (Pappalardi et al., 2021), should provide important alternatives for therapeutic applications. And although a strong reduction in DNA methylation was shown to cause genetic instability and apoptosis (Jackson-Grusby et al., 2001; Eden et al., 2003; Chen et al., 2007), partial inhibition by the highly selective small-molecule DNMT1 inhibitor GSK3685032 showed good *in vivo* tolerability (Pappalardi et al., 2021). In agreement with this notion, mice that carry a heterozygous null allele for Dnmt1 are viable and fertile, without any detectable phenotypic aberrations (Li et al., 1992). While additional work will be required to precisely define the therapeutic window for nutraceutical or cosmetical applications of DNA methylation inhibitors, exposure to DHM resulted in substantially more moderate changes in DNA methylation patterning compared to some of the established DNA methylation inhibitors. These results indicate a considerable epigenetic safety of DHM. This, and the good tolerability of DHM represent distinctive features that support the use of the compound in nutraceutical and cosmetical applications.

Our results also show that DHM reduces the DNA methylation age in cultured primary human keratinocytes. We confirmed the DHM-dependent reduction of DNA methylation age with two independent clocks, with the newly trained human epidermis clock showing a slightly stronger effect on the DNA methylation age than the well-established Skin & Blood clock (Horvath et al., 2018). This difference may be because we trained our algorithm only on epidermis samples to achieve improved prediction accuracy. Importantly, this finding adds DHM to a growing list of active substances that have a measurable impact on the biological age of human cells and tissues (Fahy et al., 2019). Furthermore, our analysis revealed an association between DNA methylation age and two key phenotypic features of human skin aging, visual appearance, and skin elasticity, independent of the chronological age. These findings are consistent with the notion that a reduction in the epigenetic age is indicative of an improved functionality of human tissues and organs (Horvath and Raj, 2018). This notion is further supported by the results obtained with our recently developed wrinkle predictor, which links the DNA methylation pattern to the arguably most relevant skin aging phenotype (see accompanying manuscript by Bienkowska et al.).

Interestingly, the moderate DNMT1 inhibition by DHM was associated with a measurable rejuvenating effect on the level of DNA methylation and gene expression *in vivo*. The vast majority of differentially methylated CpGs revealed a methylation pattern that was similar to the young epidermis. Although DHM was originally identified as a DNMT1 inhibitor and subsequently shown to induce moderate global hypomethylation *in vitro*, hypo-as well as hypermethylated CpGs were observed after the 2-weeks of topical DHM treatment *in vivo*. This can be explained by secondary epigenetic effects leading to re-methylation of distinct CpGs and/or through a reset of the genetically encoded methylation pattern. For example, our data indicate that the expression of *TET2* was upregulated in keratinocytes after DHM treatment (Supplementary Figure S10). TET2 is an epigenetic enzyme which antagonizes DNMTs to shape methylation patterns (Lyko, 2018). In addition to TET2, the expression of two other epigenetic modifiers, *KDM5C* and *PRDM2*, was also upregulated upon DHM treatment in keratinocytes (Supplementary Figure S10). While KDM5C demethylates histones H3K4 and H3K9, PRDM2 methylates histone H3K4. The upregulation of both enzymes may lead to downstream changes in H3K4 and H3K9 methylation patterns, which can influence the binding of DNMTs, and consequently DNA methylation (Li et al., 2021). Further research will be required to determine the mechanisms of DHM-associated CpG hypermethylation in detail.

In agreement with the rejuvenating effects observed at the level of DNA methylation, topical DHM treatment also had a measurable effect on age-related silencing of gene expression *in vivo*. This effect was observed for genes from various pathways, indicating a broad impact across various skin aging-related processes and several of the analyzed marker genes were related to the skin aging phenotype of wrinkle formation. For example, LYPD3 acts on cell-cell adhesion and has been described to play a role in epidermis differentiation and stratification (Jacobsen et al., 2017). In addition, our studies showed an association between reduced expression of LYPD3 with increased wrinkle development in human skin. Also, HAS3 expression is related to wrinkle formation and beside HAS1 it is suggested to play a crucial role in the regulation of hyaluronic acid synthesis in the epidermis (Sayo et al., 2002), thus being important for keratinocyte differentiation, as hyaluronic acid is involved in the formation, organization, and stabilization of the epidermal extracellular matrix (Malaisse et al., 2014). SPON2 is modulating cell communication between keratinocytes and fibroblasts, as it has been shown to be secreted by keratinocytes and induces fibrogenesis (Evrard et al., 2021). IRS2 is important for adequate wound healing (Lima et al., 2012; Manzano-Nunez et al., 2019), which is delayed in elderly skin (Amini-Nik et al., 2022) and loss of EDA is associated with dry skin and eczema (Kowalczyk-Quintas et al., 2015), which are more frequently observed in elderly people (White-Chu and Reddy, 2011). Loss of SLC25A25 is associated with a reduced basal mitochondrial respiration and ATP content in mice (Anunciado-Koza et al., 2011), which represents another key hallmark of human skin aging (Wei et al., 2009; Schniertshauer et al., 2018) and our studies indicate an association with increased wrinkle development in human skin.

Restoring the regulatory landscape of an aged cell could thus have a more comprehensive impact on the multidimensional process of aging than targeting single factors or pathways. This hypothesis was further supported by our studies using an organotypic skin culture. 3-dimensional skin models capture the complex process of skin aging better than 2-dimensional cell culture models. The robust dermal compartment, created by fibroblasts grown within an organized extracellular matrix, supports the development of a differentiated, stratified, and keratinized epidermis, with spatiotemporal cellular communication and barrier properties (Costello et al., 2022). By integrating cell populations from older donors intrinsic aging is simulated and skin equivalents exhibited many aspects of the ageing phenotype including epidermal atrophy and reduced organization of the epidermal strata (Ahlers et al., 2022). Hence, the 3D model is suitable to investigate holistic anti-aging effects, specifically for the human skin. Interestingly, using such a 3-dimensional aging model, we observed a DHM-dependent transformation of the epidermal structure towards a more juvenile, which further establishes DHM as an interesting natural compound that links epigenetic modulation to skin rejuvenation. Future studies will be required to investigate whether DHM also improves healthy skin aging *in vivo*, either as a standalone application or in combination with healthy lifestyles.

## Materials and Methods

### Ethic approval

Ethical approval was obtained in consideration of the Declaration of Helsinki and the guideline of the International Conference on Harmonization Good Clinical Practice (ICH GCP) by the International Medical & Dental Ethics Commission in Hamburg (Std. no. 67686) and by the Independent Ethics Committee Freiburg (feki code 08/2610).

### Production of DNMT1 protein

Full-length human DNMT1 protein was produced and purified as described before (Castellano et al., 2008). Briefly, the protein was expressed in insect cells and purified by affinity chromatography and gel filtration. The protein concentration of purified DNMT was determined by Bradford assay and verified by using Coomassie stained SDS/polyacrylamide gels and suitable molecular mass markers of known concentration.

### Compound screening

A homogenous 384-well scintillation proximity assay (SPA) was developed and optimized for screening of DNMT-1 inhibitors. In this assay ^3^H-SAM (PerkinElmer) is used as a methyl-group donor in the methylation of a biotinylated 42mer hemi-methylated substrate comprising the following two oligonucleotides (IBA): 5′ (bio-LC)GAT CCG ACG ACG ACG ACG AXG ACG ACG ACG ACG ACG ACG ATC (X = 5 methylcytosine) and 5′GAT CGT CGT CGT CGT CGT CGT CGT CGT CGT CGT CGT CGG ATC. The methylated is captured with yttrium silicate (YSi) streptavidin coated SPA beads (PerkinElmer). The library containing the subsets: FDA approved drugs (Enzo Life Sciences), Naturstoffe (Enzo Life Sciences), Natural products (Cfm Tropitzsch), Flavonoid derivatives (TimTec) and a collection of additional compounds (Beiersdorf AG) was screened on two separate experimental days against recombinant human DNMT1 at the DKFZ/EMBL Chemical Biology Core Facility. Compounds/substances were diluted 1:25 in H_2_O and dispensed into 384-well Optiplates. 800 nM ^3^H-SAM was added to 100 nM 42mer oligo and 30 nM DNMT1 to start the methylation reaction (180 min at room temperature). Subsequently, YSi beads were added with 372 mM NaCl to stop the methylation reaction and the plates were read on a MicroBeta LumiJet.

### Biochemical DNMT assay

Myriceline™ SPE90 extract from *Myrica cerifera* (>95% DHM, Provital, “DHM1”) and two dihydromyricetin extracts from *Ampelopsis grossedentata* (>95% DHM, Naturalin “DHM2”, BOC Sciences “DHM3”) were dissolved in DMSO. DNMT inhibition was tested using the DNMT Activity Quantification Kit (Abcam) following the manufacturer’s instructions. 0.1% DMSO was used for controls.

### DNA methylation analysis of primary human keratinocytes

Primary human keratinocyte lines from 13 independent donors were cultivated for 3 days in KGM Gold media (Lonza) supplemented with 20 µM DHM (Provital) in DMSO (final concentration: 0.1%). Genomic DNA was isolated with the QIAamp DNA Investigator Kit (Qiagen) and processed for Methylation EPIC arrays (Illumina). Methylation data analysis was carried out using the R Bioconductor package *Minfi* (Aryee et al., 2014) and *limma* (Ritchie et al., 2015). Specifically, raw. idat files were read and preprocessed. Methylation loci (probes) were filtered for high detection *p*-value [*p* > 0.01, as provided by *Minfi* (Aryee et al., 2014)], location on sex chromosomes, ability to self-hybridize, and potential SNP contamination. Array normalization was performed using the *preprocessFunnorm* function, available in *Minfi* (Aryee et al., 2014). Quality control was performed after every preprocessing step. Subsequently, differentially methylated probes were identified by fitting a linear model with a paired sample design followed by statistical analysis using an empirical Bayes method to moderate standard errors. Lastly, differentially methylated probes were filtered by significance threshold (P_adj_ < 0.05, *t*-test, after correction for multiple testing using the Benjamini–Hochberg method).

Additionally, we trained a classifier to discriminate between DHM-treated and DMSO control cells using a machine learning approach. This analysis was performed using the *randomForest* function provided by the *randomForest* R package (Liaw and Wiener, 2002). Training was performed using the differentially methylated probes from the previous analysis, with the treatment status of the samples converted into a factor variable. The random forest classifier was built with 500 trees, and 10-fold cross-validation was applied.

Primary human keratinocyte lines from 2 independent donors and 2 keratinocyte cell lines were cultivated for 3 days in KGM Gold media (Lonza) supplemented with 20 µM DHM (Provital) in DMSO (final concentration: 0.1%). Genomic DNA was isolated with the QIAamp DNA Investigator Kit (Qiagen) and LINE-1 promotor methylation (sequence to analyze: TTYGTGGTGYGTYGTTTTTTAAGTYGGTTTGAAAAG) was analyzed by EpigenDX using direct pyrosequencing after bisulfite conversion.

### Training of DNA methylation clock and methylation analysis

We made use of a methylation dataset (Methylation EPIC arrays, Illumina) obtained from epidermis samples of the population-based Study of Health in Pomerania (SHIP-TREND-1) (Volzke et al., 2022) comprising 461 female participants. The data were processed as described previously (see section analysis of primary human keratinocytes) using quantile normalization (*preprocessQuantile*). Before training, one probe (cg26614073) was excluded from the dataset to avoid conflict with Horvath’s patent claims (EP 3 049 535 B1). After splitting 378 females of the dataset (80% for training and 20% for testing), the training of the age clock was conducted with the beta-values of the training dataset applying the *cv. glmnet* function (alpha = 0, lambda = 1408.78) of the *glmnet* R package (Friedman et al., 2010) in a 10-fold cross-validation mode. The trained clock was validated with the test set and an independent publicly available dataset (Holzscheck et al., 2020a) (Supplementary Figure S5A) and outperformed the Skin & Blood clock established by (Horvath et al., 2018) (Supplementary Figure S5B). The methylation levels of the independent dataset were obtained and pre-processed in the same way as the dataset used for the training. Finally, the obtained DNA methylation clock was used to determine the methylation age of the primary cultured keratinocytes. Additionally, the methylation age of the primary cultured keratinocytes was determined using the published Skin & Blood clock (Horvath et al., 2018). Finally, the predicted wrinkle grade of the primary cultured keratinocytes was determined using our recently developed wrinkle predictor (see accompanying manuscript by Bienkowska et al.).

### Correlation of skin aging phenotype and DNA methylation age

We used the methylation and phenotypic data from epidermal samples of SHIP-TREND-1 (Volzke et al., 2022). We calculated the DNA methylation age via a published skin specific DNA methylation clock (Bormann et al., 2016) to make use of the whole dataset, as the published clock was trained on data from another study. For the same study participant their visual age was determined as the average rated age appearance based on the volunteer’s portrait pictures and assessed by more than 30 experts. Additionally, before epidermal samples were taken, the skin elasticity on the sample area was measured for each study participant using a cutometer^®^ (Courage & Khazaka Electronic GmbH, Cologne, Germany) as previously described (Dobrev, 2002). The elasticity was defined as the average of 3 consecutive measurements of the R7 value. Partial correlation analysis was performed as follows: First, the residuals of the linear regression between the parameters of interest and the chronological age were calculated (y ∼ chronological age; with y = methylation age, visual age, and skin elasticity, respectively). Subsequently, the association between the residuals was analyzed by Pearson correlation analysis to determine the significance of the correlation.

### *In vivo* DNA methylation analysis

The skin of the lower back from 30 female volunteers was treated for 2 weeks by topical application with a formulation based on oil-in-water emulsions (o/w) containing 0.15% DHM (w/w) or vehicle, followed by repetitive UV exposure at a sub-erythemal dose (Holzscheck et al., 2020b) as an aging stimulus. 24 h after the last UV exposure, suction blister samples were obtained (Südel et al., 2003), DNA was isolated and DNA methylation patterns were analyzed using Infinium Methylation EPIC arrays (Illumina). Data were processed using *Minfi* (Aryee et al., 2014) and quantile normalization was applied. For the subsequent analyses M values were used to describe CpG methylation levels. CpGs were considered significantly differentially methylated, if the adjusted *p*-value was <0.05 using the Benjamini–Hochberg procedure. Data analysis revealed 16,389 CpGs, which were specifically differentially methylated in a DHM-dependent manner on the irradiated skin area. In parallel, we used the methylation data from the big cohort study (see section training of DNA methylation clock and methylation age analysis) analyzing the methylation patterns from 102 younger (28–40 years) and 102 older volunteers (65–88 years) and we found 270,642 probes to be differentially methylated in aged samples. The intersection of the 16,389 DHM-dependent probes with the 270,642 age-dependent probes defined the set of 12,444 DHM-dependent and age-dependent probes.

### *In vivo* gene expression analysis

The skin of the inner forearm from 19 female volunteers (50–65 years) was treated for 8 weeks by topical application with a formulation based on oil-in-water emulsions (o/w) containing 0.15% DHM (w/w) or vehicle. After completion of the treatment, suction blisters were obtained for RNA isolation. In parallel, we analyzed gene expression datasets (RNA-Seq) obtained from epidermis samples of SHIP-TREND-1 (Volzke et al., 2022), which were analyzed with the R Bioconductor package *DESeq2* (Love et al., 2014). This identified 2,820 genes which were downregulated (logFC <0, P_adj_ ≤ 0.05) in the epidermis of older volunteers (≥70 years) compared to the younger group (≤40 years) and which were associated with hypermethylated CpGs. The expression of 34 of these genes (selected based on keratinocyte-relevant pathway association) was analyzed using qRT-PCR. To this end, total RNA of suction blister roofs was isolated using the RNeasy Fibrous Tissue Mini Kit (Qiagen) according to the manufacturer’s protocol. After reverse transcription using the High Capacity cDNA Reverse Transcription Kit (Applied Biosystems) gene expression was detected using the Real-Time TaqMan PCR (Applied Biosystems). Ct values were normalized to endogenous housekeeping gene 18sRNA and gene expression fold changes were calculated via the 2^−ΔΔCt^ method.

### 3D skin model and histology

Three-dimensional skin models were generated as previously described (Boehnke et al., 2007). Hyalograft-3D was replaced by the non-woven fibrous scaffold M3-II Bemcot (Asahi Kasei, Tokyo, Japan). After 6 weeks of culture, DHM at a final concentration of 20 µM dissolved in DMSO (0.1%) was added to the medium for additional 6 weeks. Sections of 3D skin equivalents were stained using AEMA fast staining Kit (LT Lehmann, Berlin, Germany) according to manufacturer’s protocol and epidermal thickness was determined using Fiji software (Schindelin et al., 2012).

### Crosstalk experiment

Fibroblasts were seeded in 96-well plates at a density of 5 × 10^3^ cells/well in DMEM (Gibco) with 10% FCS (Biowest) and 1% Penicillin/Streptomycin (Sigma-Aldrich) and keratinocytes were seeded in six-well-plates at a density of 5 × 10^3^ cells/well in KGM Gold (Lonza). Keratinocytes were treated with 39 µM DHM for 24 h. Keratinocyte-conditioned media were filtered (Sartorius Vivaspin Turbo4 10,000 MW Cutoff) and retentates were mixed with fresh KGM and transferred to fibroblasts. After 24 h fibroblasts were PFA-fixed, stained (EarlyTox DeadGreen, Molecular Devices) and imaged (SpectraMax, Molecular Devices).

### DNA damage assay

Primary human keratinocytes were seeded in 96-well plates at a density of 10 × 10^3^ cells/well in KGM Gold (Lonza) and preincubated overnight at 37°C/5% CO_2_. Cells were then treated with 10 μM and 20 µM DHM for 72 h at 37°C/5% CO_2_. DNA double strand breaks were induced in positive control wells with 250 µM hydrogen peroxide (AppliChem) for 15 min. Cells were then fixed with formaldehyde followed by immunostaining with Anti-Phospho-H2A.X-AlexaFluor^(^™^)^555 antibody (Merck Millipore) and counterstaining with Hoechst 33,342 (ThermoFisher). Image acquisition and analysis was performed on the semi-automated high-content imaging system scanR (Olympus).

### Apoptosis assay

Primary human keratinocytes were seeded in 96-well plates at a density of 10 × 10^3^ cells/well in KGM Gold (Lonza) and preincubated overnight at 37°C/5% CO_2_. Cells were treated with 10 μM and 20 µM DHM for 72 h at 37°C/5% CO_2_. Caspase 3/7 activity was assessed using the Caspase 3/7-Glo luminescence assay (Promega).

### Safety assessment

Safety assessment followed next-generation risk assessment approaches for new cosmetic ingredients without animal testing. These combine any existing and relevant data from *in vivo* animal studies with data generated with *in vitro* assays, *in silico* predictions, read-across analyses and other biological or computational models (Alexander-White et al., 2022; Westmoreland et al., 2022) to cover toxicological endpoints such as acute toxicity, skin irritation, skin sensitization, genotoxicity and systemic toxicity.

### Phototoxicity assay

To determine the phototoxicity of UV absorbing compounds the Phototoxicity Test according to the OECD Guideline 498 was performed using the human reconstructed epidermis model EpiDerm™ (MatTek; *InVitro* Life Science Laboratories, s. r.o.) according to the previously described protocol (Kandarova and Liebsch, 2017). Briefly, DHM was applied topically to epidermal surface, 24 h later the skin equivalents were exposed to UV irradiation (Hönle SOL 500; applied dosis: 1.7 mW/cm2 für 60 min = 6 J/cm2). After additional 24 h cytotoxicity has been evaluated by reduction of mitochondrial conversion of MTT to formazan (MatTek; *InVitro* Life Science Laboratories, s. r.o.).

### Cell viability assay

To determine the most tolerable *in vitro* concentration of DHM, we measured endogenous esterase activity. Briefly, keratinocytes of three different donors were cultured in 96-well plates at a density of 10 × 10^3^ cells/well in KGM Gold (Lonza) for 72 h in the presence of 0.125µM–160 µM DHM. Subsequently, cells were washed with 1× Dulbecco’s Phosphate Buffered Saline (DPBS) and incubated for 15 min at 37°C in 100μL/well fluorescein diacetate (FDA) (Sigma, Taufkirchen, Germany) solution (15 μg/mL FDA in 1 × DPBS). Fluorescence was then determined at 517 nm in the 96-well plate reader infinite M200 (Tecan, Crailsheim, Germany).

### *In vitro* gene expression analysis

To determine if DHM stimulates gene expression of epigenetic regulators in skin cells, human primary epidermal keratinocytes were isolated from skin biopsies obtained from six different donors (55–72 years) as described previously (Reichert et al., 2017). After isolation, keratinocytes were seeded in six-well plates (7.5 × 10^4^ cells/well) and cultured in KGM-Gold (Lonza) for 24 h. In the following, cells were treated with 20 µM DHM for 72 h. Total RNA was then extracted using the RNeasy Mini Kit (Qiagen) according to the manufacturer’s protocol. After hybridization using target-specific Reporter and Capture probes, reactions were purified and imaged on the nCounter system (NanoString). Raw mRNA counts were normalized using five endogenous housekeeping genes (GAPDH, GUSB, OAZ1, PUM1, UBC) and six NanoString positive controls that were spiked into every sample. Fold Change values were calculated as the ration of normalized mRNA counts (DHM vs. 0.1% DMSO treatment).

### Correlation of wrinkle grade and gene expression

To further demonstrate the relevance of the selected age-related epigenetically silenced genes for the skin aging phenotype, we analyzed the relation between their expression and the grade of *in vivo* observed wrinkle formation. Like for the correlation analysis of the skin aging phenotype with the DNA methylation age, we made use of the data from the SHIP-TREND-1 study (Volzke et al., 2022). To ensure generalizability of the results, we used four different approaches to represent the gene expression for the correlation analysis: i) raw read counts, ii) transcripts per million (TPMs), iii) normalized read counts and iv) normalized TPMs. Normalization was performed in analogy to RT-PCR approaches by dividing the gene expression of each gene by the geometric mean of the housekeeping genes (*GAPDH*, *GUSB*, *OAZ1*, *PUM1* and *UBC*). The Pearson correlation between gene expression and wrinkle grade was determined with the *cor. test* function in R and the calculated *p*-values were then adjusted for multiple testing with the R function *p. adjust*. Finally, only the expression of genes which had an adjusted *p*-value <0.05 in all four approaches were considered as significantly correlating with the wrinkle grade.

## Data Availability

The data of the cohort study are under special EU privacy legislation due to their sensitive nature and hence, only available from the SHIP consortium upon official request. The datasets analyzed for this study can be found in the Array Express database https://www.ebi.ac.uk/biostudies/arrayexpress via E-MTAB-8993.
