# Supplementary material for: Identification of dihydromyricetin as a natural DNA methylation inhibitor with rejuvenating activity in human skin
Source: Front Aging. 2024 Mar 4;4:1258184. doi: 10.3389/fragi.2023.1258184 (PMC10944877; doi:10.3389/fragi.2023.1258184)
Supplement: Supplementary file 1 [file Table1.pdf]

Supplementary Material

Identification of Dihydromyricetin as a natural DNA methylation inhibitor with rejuvenating activity in human skin

Cassandra Falckenhayn, Agata Bienkowska, Jörn Söhle, Katrin Wegner, Günter Raddatz, Boris Kristof, Dirk Kuck, Ralf Siegner, Ronny Kaufmann, Julia Korn, Sascha Baumann, Daniela Lange, Andreas Schepky, Henry Völzke, Lars Kaderali, Marc Winnefeld, Frank Lyko, Elke Grönniger*

*** Correspondence:** Elke Grönniger: elke.groenniger@beiersdorf.com

1 Supplementary Figures

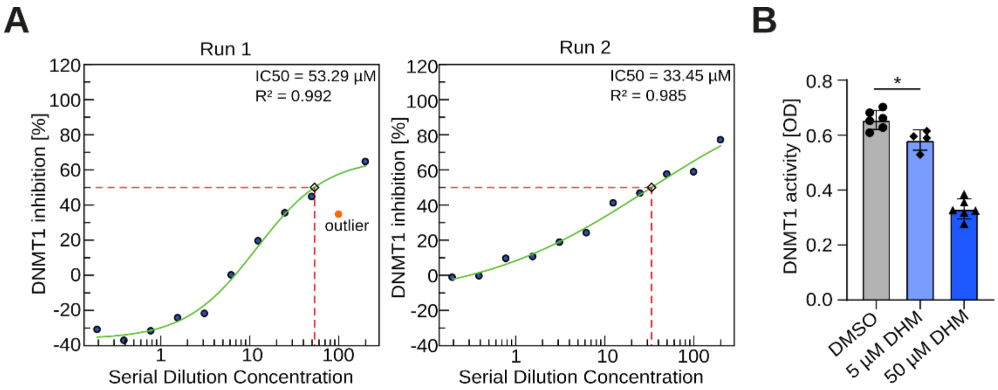

Supplementary Figure S1. Concentration dependent inhibition of DNMT1. (**A**) IC_{50} curves of myricetin from the two independent screens. The corresponding IC_{50} and the R^2 of the fitted line are indicated in the plot. (**B**) Independent biochemical DNMT assay testing 5 µM (N = 4) and 50 µM (N = 6) DHM (*P = 0.0125 unpaired t-test).

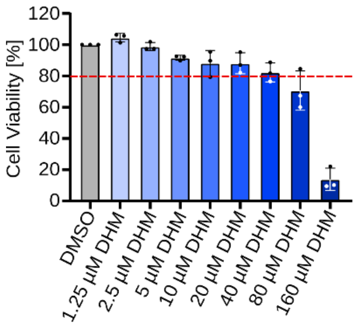

Supplementary Figure S2. Keratinocyte viability after DHM treatment. Fluorescein Diacetate Assay (FDA) of keratinocytes was conducted after DHM treatment. Cell viability of 80% is indicated by a red dashed line. Concentrations of up to 20 µM DHM showed a good tolerability (N=3).

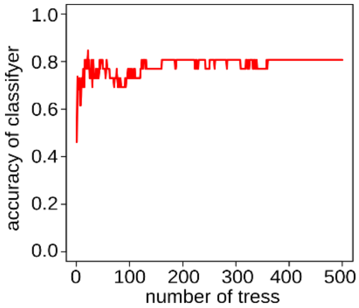

Supplementary Figure S3. Accuracy during training epochs (number of trees) of the random forest classifier in discriminating between DMSO and DHM treated primary human keratinocyte cells based on the 101,067 differentially methylated probes. The average accuracy of the trained 10-fold cross-validated model is 78.43%.

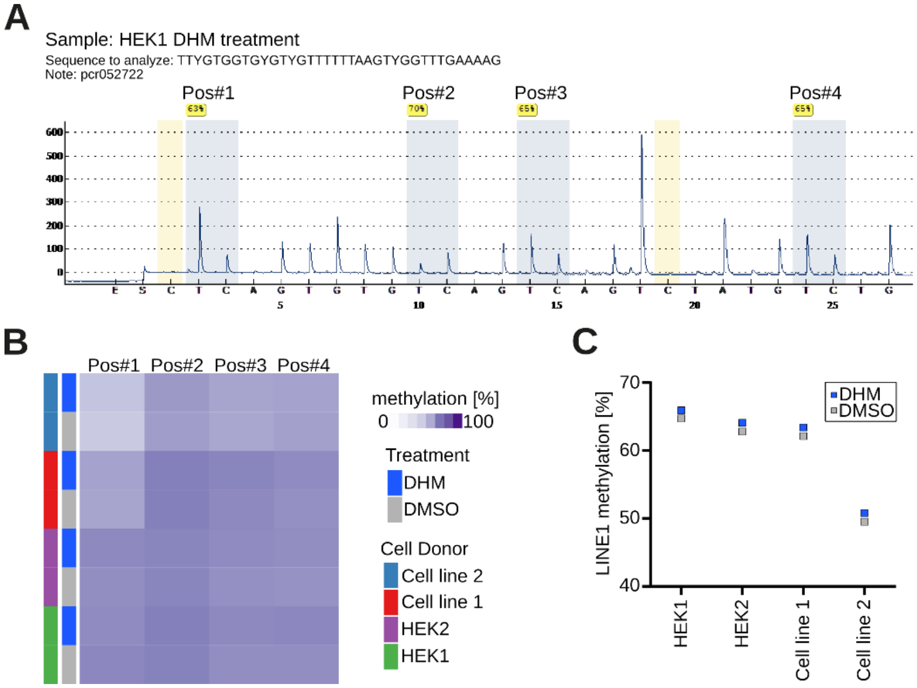

Supplementary Figure S4. LINE-1 methylation analysis. (**A**) Representative example of the CpG pyrogram report of the sequenced LINE-1 element after DHM-treatment. Positions of methylation are highlighted in blue and positions serving as control for bisulfite conversion are highlighted in yellow. (**B**) Heatmap summarizing the percent methylation at each LINE-1 methylation site for the DHM-treatment (blue bar) in context to the control DMSO-treatment (gray bar) for each of the four experiments; two primary human keratinocytes (HEK1 and HEK2) and two keratinocyte cell lines (cell line 1 and cell line 2). (**C**) Visualization of the average methylation of the LINE-1 element between control (gray) and DHM treatment (blue) for each experiment.

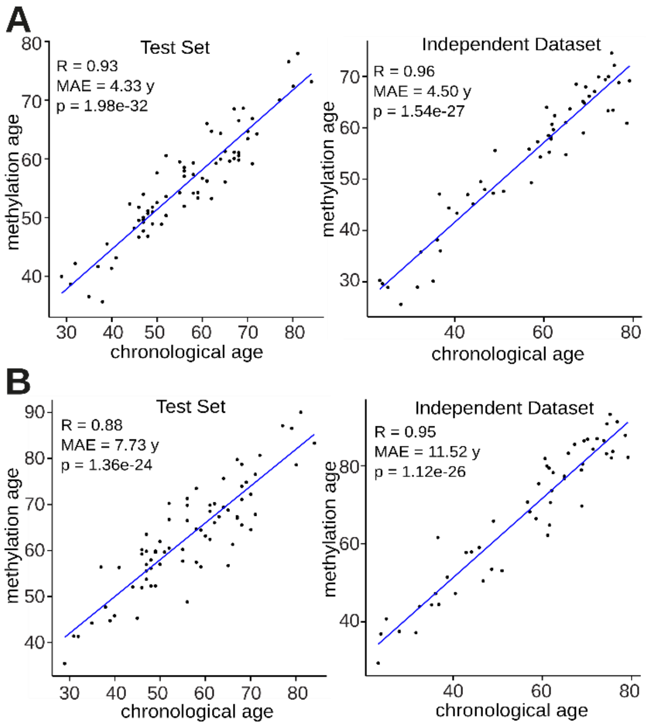

Supplementary Figure S5. Validation and establishment of the newly trained DNA methylation clock. (**A**) Performance of the trained clock of this publication on the test dataset and on an independent dataset from (Holzscheck et al., 2020a). (**B**) Performance of the Skin & Blood DNA methylation clock (Horvath et al., 2018) on the same datasets. Each panel reports the Pearson correlation and the mean absolute error.

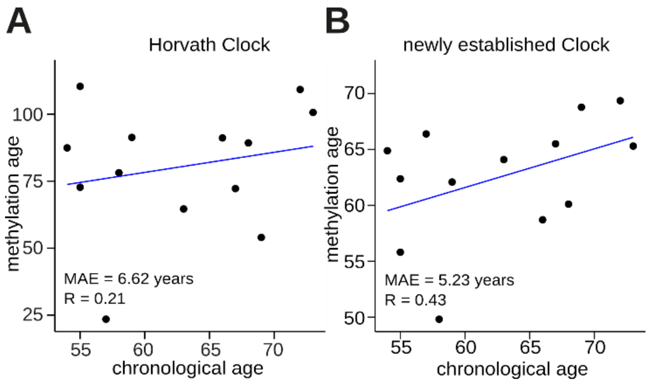

Supplementary Figure S6. Correlation between donor age and predicated biological age of the in vitro cultured primary keratinocytes. The biological age was determined by applying the methylation data of the cell culture samples to the (**A**) established Skin & Blood Clock (Horvath et al., 2018) and (**B**) the newly established clock. The correlation coefficient R^2 and the mean absolute error (MAE) are depicted for each clock.

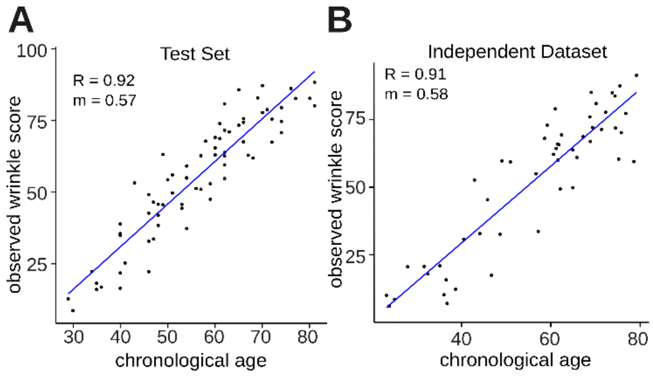

Supplementary Figure S7. Correlation between chronological age and observed wrinkle score in datasets of (**A**) (Volzke et al., 2022) and (**B**) (Holzscheck et al., 2020a).

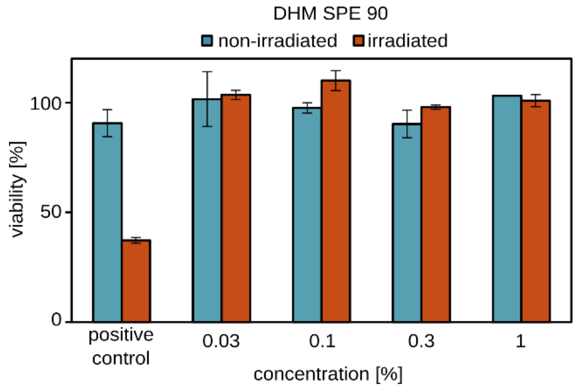

Supplementary Figure S8. Phototoxicity Test. Viability of topical DHM treatment of human reconstructed epidermis models with (orange) and without (turquoise) UV irradiation (1.7 mW/cm^2 für 60 min = 6 J/cm^2). For comparison the phototoxic effect of a positive control is depicted on the two left bars.

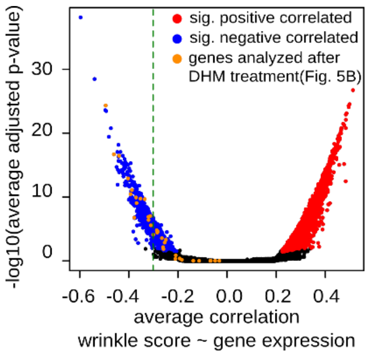

Supplementary Figure S9. Correlation between gene expression and wrinkle grade. Volcano plot depicts the averaged correlation coefficient (x-axis) against the averaged adjusted p-value (y-axis) among the four correlation approaches (see Material and Methods for details). Genes with significant negative correlation in all four analyses are highlighted in blue and genes with a significant positive correlation in red ($P_{adj}<0.05$, Pearson correlation with Holm correction). Genes which have been investigated after DHM treatment in vivo (in Figure 5B) are highlighted in orange. The green dashed line indicates the border of the top 10 % of genes significantly negatively correlating with wrinkle grade.

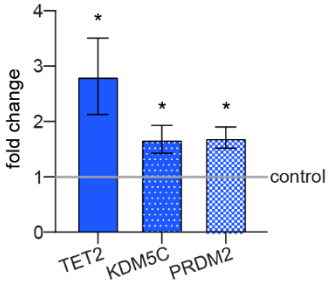

Supplementary Figure S10. Upregulation of the methylcytosine dioxygenase TET2, H3K4/K9-demethylase KDM5C and H3K4-methyltransferase PRDM2 after DHM treatment in primary human keratinocytes. Gene expression of the three epigenetic modifier was analyzed by qPCR in 6 primary keratinocyte lines (N=6) from independent donors after 3 days of DHM treatment. *P<0.01, t-test. Values are relative to DMSO control (=1).

2 Supplementary List

Supplementary List S1. List of abbreviation used in the manuscript.

**Abbreviation**	**Description**
18S rRNA	18 Svedberg ribosomal RNA
3D	three dimensional
ADIPOR	Adiponectin receptor
AGPAT	Glycerol-3-phosphate acyltransferase
ALM	acute myeloid leukemia
AMN	Amnionless
ANOVA	analysis of variance
AOP	Anterior open
ATP	adenosine triphosphate
cDNA	complementary DNA
CH25H	cholesterol 25-hydroxylas
CLDN	claudin
CO2	carbon dioxide
COL6A1	collagen type VI alpha-1 chain
Conc.	concentration
CpG	cytosine guanine dinucleotide
CPM	counts per million
Ct-value	cycle threshold value
DAC	Decitabine
DDSB	DNA double strand breaks
DHM	dihydromyricetin
DKFZ	Deutsches Krebsforschungszentrum
DMEM	Dulbecco's Modified Eagle's Medium
DMSO	dimethyl sulfoxide
DNA	Deoxyribonucleic acid
DNMT	DNA methyltransferase
DPBS	Dulbecco's Phosphate Buffered Saline
ECM	extra cellular matrix
EDA	Ectodysplasin A
ELF	acute myeloid leukemia cell line
EMBL	European Molecular Biology Laboratory
ERRFI1	ErbB receptor feedback inhibitor
FCS	fetal calfs serum
FDA approved	Food and Drug Administartion
FDA assay	Fluorescein Diacetate Assay
FOXO	Forkhead-Box-Protein
FZD	Fizzled
gamma-H2AX	H2A histone family member X
GAPDH	Glyceraldehyde 3-phosphate dehydrogenase

GENCODE	Genome ENCyclopedia Of DNA Elements
GEO	Gene Expression Omnibus
GSK	GlaxoSmithKline
GUSB	β-glucuronidase
H2O	Water
H2O2	hydrogen peroxide
H3K4	foruth lysine residue on histone H3
H3K9	nineth lysine residue on histone H3
HA	hyaloronic acid
HAS	hyaluronan sythase
HEK	human epidermal keratinocytes
IC50-value	half maximal inhibitory concentration
ICH GCP	International Conference on Harmonization Good Clinical Practice
idat	itensity data file
IL6R	interleukin-6 receptor
IRS	insulin receptor substrate
ITGA	integrin alpha
ITGB	integrin beta
KDM5C	Lydine-specific demethylase 5C
KGM	keratinocyte growth medium
LDLR	low-density lipoprotein receptor
LINE	long interspersed nuclear elements
logFC	the logarithm of the fold change to base 2
logP	the logarithm of the partition coefficient P
LPIN1	Lipin
LYPD	Ly6/PLAUR domain-containing protein
MAE	mean absolute error
MA-plot	log ratio (M) plotted against average mean (A)
mRNA	messenger RNA
MTT	dimethyl thiazolyl diphenyl tetrazolium salt
M-value	log ratio of methylated to unmethylated probes
Myr	myricetin
NaCl	natrium chloride
NEDD4L	Neural precursor cell expressed developmentally downregulated gene 4-like
NOS	nitric oxide synthase
OAZ	Ornithine decarboxylase antizyme
OD	optical density
ODC	ornithine decarboxylase
OECD	Organisation for Economic Co-operation and Development
Padj	adjusted P-value
PFA	paraformaldehyd
PI4KB	Phosphatidylinositol 4-kinase beta
PLIN	perilipin
PRDM	PR domain zinc finger protein

pred.	predicted
PUM1	Pumilio homolog 1
P-value	probability value
qRT-PCR/ qPCR	quantitative real-time polymerase chain reaction
R7-value	ratio of immediate reaction to total deformation
RHPN	Rhophilin
RLU	relative light units
RNA	ribonucleic acid
RNA-Seq	RNA sequencing
SAH	S-adenosyl-homocysteine
SAM	S-adenosyl-methionine
SDS	sodium dodecyl sulfate
SHIP	Study of Health in Pomerania
sig.	significant
SLC25A25	Solute Carrier Family 25 Member 25
SLCO4A1	Solute carrier organic anion transporter family member 4A1
SPA	scintillation proximity assay
SPE90	Myriceline extract name
SPON	Spondin
SPTLC2	Serine palmitoyltransferase, long chain base subunit 2
STX	Syntaxin
TE	transposable element
TET	ten-eleven translocation methylcytosine dioxygenase
TPM	transcripts per million
t-test	hypothesis test statisitc
TUFT	Tuftelin
UBC	Polyubiquitin-C
UV	ultraviolet
VCL	vinculin
VPS37B	ESCRT-I subunit
wri. sc.	wrinkle score
YSi	yttrium silicate
